# Supplementary material for: Cellular HIV Reservoirs and Viral Rebound from the Lymphoid Compartments of 4′-Ethynyl-2-Fluoro-2′-Deoxyadenosine (EFdA)-Suppressed Humanized Mice
Source: Viruses. 2019 Mar 13;11(3):256. doi: 10.3390/v11030256 (PMC6466357; doi:10.3390/v11030256)
Supplement: Supplementary file 1 [file viruses-11-00256-s001.pdf]

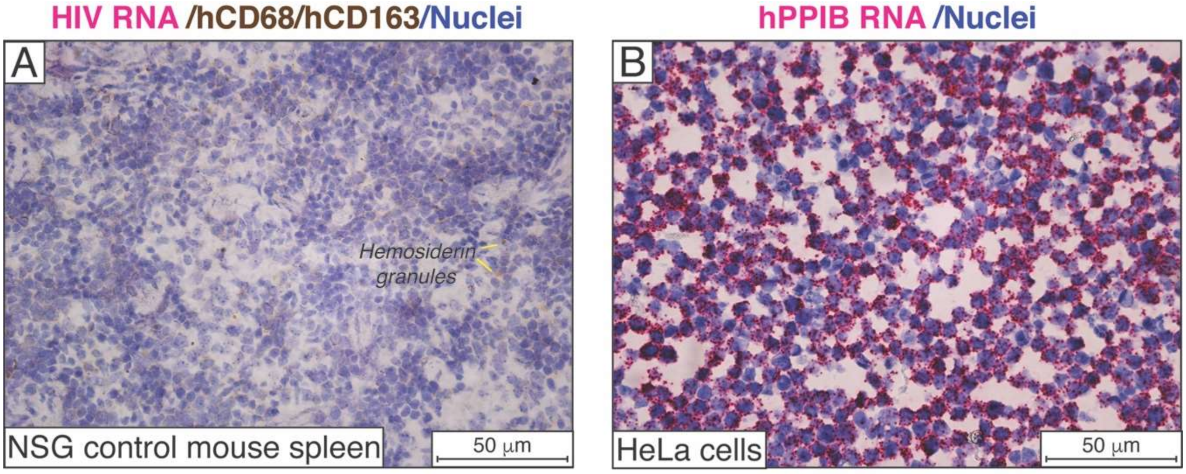

Figure S1. HIV detection in control tissues**.** HIV RNA and human CD163^{+}CD68^{+} macrophages were not detected in the spleen of a NSG mouse that was not implanted with human tissues (**A**), whereas high levels of human PPIB RNA expression were detected in HeLa cells (**B**). Nuclei were counterstained with hematoxylin.

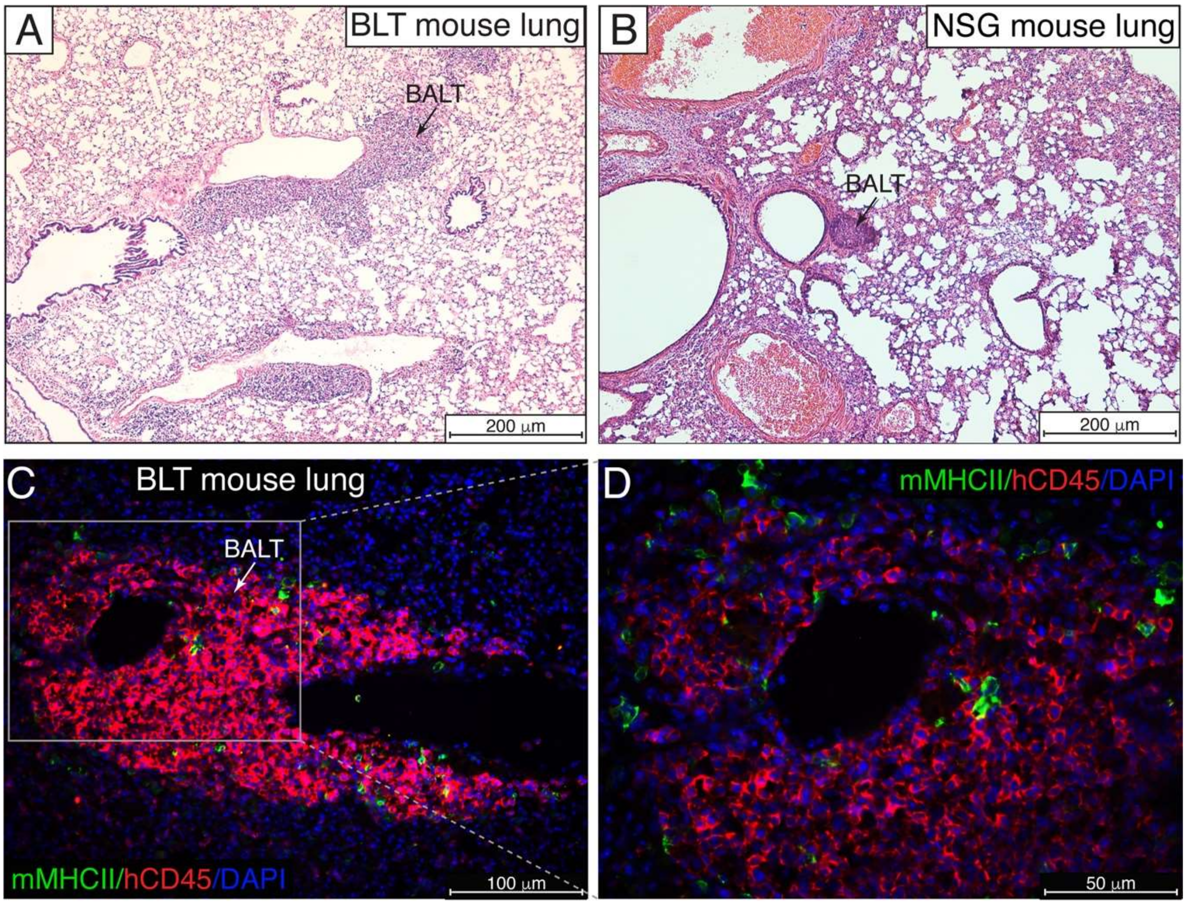

Figure S2. NSG-BLT mouse lungs frequently contain large compartments of human lymphoid cells. Hematoxylin and eosin staining of NSG-BLT mouse lung 33 weeks after implantation (**A**) and NSG mouse lung before implantation (**B**). BALT, bronchus-associated lymphoid tissue. In the NSG-BLT mouse lung, BALT appeared as a follicular accumulation of human CD45^{+} lymphocytes intermingled with mouse macrophages identified by anti-mouse MHC class II antibody (**C**, **D**). Nuclei were counterstained with hematoxylin (**A**, **B**) and DAPI (**C**, **D**).
